# Expanding the purview of wellness indicators: validating a new measure that includes attitudes, behaviors, and perspectives

**DOI:** 10.1080/21642850.2021.2008940

**Published:** 2021-12-01

**Authors:** Carolyn E. Schwartz, Brian D. Stucky, Roland B. Stark

**Affiliations:** a DeltaQuest Foundation, Inc., Concord, MA, USA; b Departments of Medicine and Orthopaedic Surgery, Tufts University Medical School, Boston, MA, USA

**Keywords:** Wellness, measurement, validation, item response theory, classical test theory

## Abstract

**Objective:**

The present study validated the DeltaQuest Wellness Measure (DQ Wellness), a new 15-item measure of wellness that spans relevant attitudes, behaviors, and perspectives.

**Design:**

This cross-sectional web-based study recruited chronically-ill patients and/or caregivers (*n* = 3,961) and a nationally representative comparison group (n = 855).

**Main Outcome Measures:**

The DQ Wellness assesses: a way of being in the world that involves seeing and embracing the good and expressing kindness toward others; engagement in one’s activities and self-care; downplaying negative thoughts that reduce one’s energy; and an ability to feel joy. Six widely used measures of physical and mental health, cognition, and psychological well-being enabled construct-validity comparisons. Item-response theory (IRT) methods evaluated reliability, factor structure, and differential item functioning (DIF) by gender.

**Results:**

The DQ Wellness showed strong cross-sectional reliability (marginal reliability = 0.89) and fit a bifactor model (RMSEA = 0.063, CFI = 0.982, TLI = 0.983). The DQ Wellness general score demonstrated construct validity, convergent and divergent validity, unique variance, and known-groups validity, and minimal gender DIF. The study is limited to addressing cross-sectional reliability and validity, and response rates are not known due to the recruitment source.

**Conclusion:**

The DQ Wellness is a relatively brief measure, taps novel content, and could be useful for observational or interventional studies.

## Main text introduction

The concept of wellness is central to broad range of research endeavors and clinical interventions. Health outcomes research not only considers clinical and economic aspects of outcomes, but also ‘humanistic’ aspects which include symptoms, quality of life (QOL), functional status, and patient satisfaction (Epstein & Sherwood, 1996). Research on resilience to life stressors and health challenges has found the following to be highly relevant to wellness: the ability to maintain mood, life purpose, satisfaction (Hartfiel, Havenhand, Khalsa, Clarke, & Krayer, 2011), and functioning (Greene, 2014; Shatté, Perlman, Smith, & Lynch, 2017); manage one’s illness (Yi-Frazier et al., 2015); and maintain optimism and purpose in life (Smith, Epstein, Ortiz, Christopher, & Tooley, 2013). Clinical interventions directed toward helping people to cope with life stressors and/or health challenges, such as mindfulness (Creswell, 2017) or coping interventions (de Ridder & Schreurs, 2001), aim to enable individuals to maintain positive affect (Moskowitz, 2011), energy (Anshel, Umscheid, & Brinthaupt, 2013), self-care (Ko & Gu, 2004), and sense of coherence (Rohani, Abedi, Omranipour, & Langius-Eklöf, 2015). Psychological research has shown the relevance to well-being indicators of having a stable self-concept that is impervious to situational challenges (Diehl & Hay, 2010).

The field of QOL research has grown in the three decades since its inception (Patrick & Deyo, 1989; Slevin, Plant, Lynch, Drinkwater, & Gregory, 1988; Spilker, 1990) in part because of a continued (re)consideration of the meaning of health. The World Health Organization’s 1948 expansive definition of health posited that *health* is a state of complete physical, mental, and social well-being and not merely the absence of disease or infirmity (World Health Organization, 1948). Recent work by Huber et al. expanded the concept of health to include the ability to adapt and self-manage in the face of social, physical, and emotional challenges (Huber et al., 2011). Ryff’s landmark work on *psychological well-being* used as a touchstone Aristotle’s idea of eudaemonia, going well beyond mental health and/or an hedonic ideal, and extending the construct to include six domains related to connection and engagement in the world, and activities that made the world a better place (Ryff, 1989; Ryff, 2014; Ryff & Singer, 2008).

**Figure 1.**
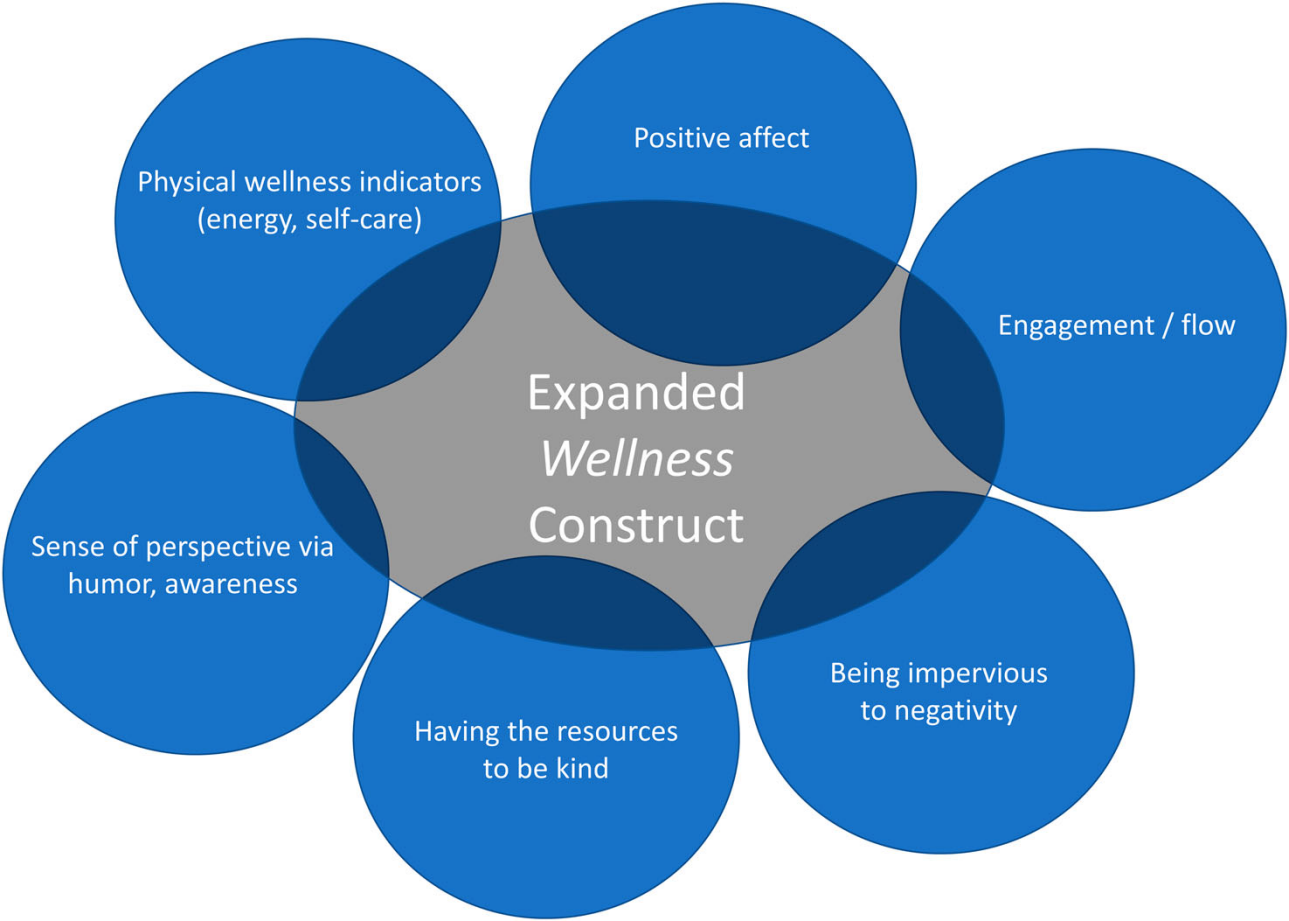
Conceptual Model of Wellness. This Venn diagram illustrates the proposed conceptual model which includes physical wellness indicators, positive affect, sense of engagement, being impervious to negativity from others, having the resources to be kind to others, and being able to retain a sense of perspective.

Accordingly, we propose extending the construct of *wellness* to extend beyond the physical-health definition of ‘good health’ in the Oxford dictionary (Simpson & Weiner, 1989), ‘quality of life’ as used in clinical research relating to patients’ multi-domain perceptions of performance (Schipper, Clinch, & Olweny, 1996), and even beyond its definition of ‘well-being’ as ‘the state of being comfortable, healthy, or happy’ (Simpson & Weiner, 1989). We propose that wellness includes attitudes, behaviors, and perspectives. Figure 1 shows a Venn diagram illustrating the proposed conceptual model. This model builds on a broad body of research. Starting at the top left of the figure and proceeding clockwise, research on health outcomes has documented that people who fare better in terms of *physical* wellness indicators such as vitality tend to follow a routine of self-care that includes diet, exercise, and sleep hygiene (Jackson & DiPlacido, 2020; Myers et al., 1999; Visser, Hirsch, Brown, Ryan, & Moynihan, 2015), even in the context of chronic illness (Riegel, Jaarsma, & Strömberg, 2012). Maintaining *positive affect* is associated with optimism (Hodges & Winstanley, 2012) and adaptive meaning-based coping (Folkman & Moskowitz, 2000). Similarly, cognitive-appraisal processes (i.e. the individual’s ways of thinking about QOL) focused on comparing him/herself to standards that shed a positive light on her/his circumstances (i.e. positive standards of comparison) and/or focus on thinking about positive aspects of her/his experience (i.e. positive tendencies in sampling of experience) have been found to be associated with better QOL outcomes among caregivers of people with haemophilia (Schwartz, Stark, Michael, & Rapkin, 2020; Schwartz, Stark, Stucky, Michael, & Rapkin, 2020) and reduced treatment burden among the chronically ill (Schwartz, Zhang, Michael, Eton, & Rapkin, 2018). Maintaining a *sense of engagement* and enthusiasm for one’s activities is associated with a sense of ‘flow’ (i.e. being so immersed in a pleasurably challenging task that time passes quickly) (Csikszentmihalyi & Csikzentmihaly, 1990), purpose in life (Ryff, 1989, 2014), and successful aging (Carstensen, Fung, & Charles, 2003). Being *impervious to negativity* from others or due to life conditions is associated with better QOL outcomes (Abravanel & Sinha, 2015; Ginandes, 2017; McNulty, 2008; Ormel & Wohlfarth, 1991), and we believe constitutes an inner fortitude that enables mood stability. This inner fortitude may relate to recognizing a consistent and underlying core ‘self’ and maintaining a sense of integrity or constancy with oneself and one’s values. Being able to recognize this core ‘self’ is relevant to depression and pharmacotherapy treatments for such. Many treatments for depression intervene effectively with dysphoria but lead to a sense of unreality or disconnection from oneself (Goodwin, Price, De Bodinat, & Laredo, 2017; Read & Williams, 2018; Read, Cartwright, & Gibson, 2014), which can undermine treatment adherence (Sansone & Sansone, 2012) and patient-reported estimates of treatment effectiveness (Hughes, Lacasse, Fuller, & Spaulding-Givens, 2017). Being *able to be kind to others* has documented associations with better health outcomes (Otake, Shimai, Tanaka-Matsumi, Otsui, & Fredrickson, 2006) and higher levels of well-being (Schwartz, Meisenhelder, Ma, & Reed, 2003; Schwartz, Quaranto, Healy, Benedict, & Vollmer, 2013). Finally, being able to retain a *sense of perspective* has been found to be an effective strategy for dealing with the loss of a loved one (Folkman, 2001; Folkman & Moskowitz, 2007). Humor is a well-documented indicator of such a perspective (Arnle, Holt, & Calhoun, 1999; Wooten, 1996), and has been found to be helpful for coping with serious illness (Cousins, 1979). Thus, this expanded concept of wellness reflects more of a ‘mind-body’ idea than a purely physical one.

Extending the construct of wellness will have implications for measurement. We acknowledge that there are many available measures of constructs related to wellness and well-being, but maintain that none of these tools capture fully the proposed construct in one brief measure. These measures include assessments of perceived wellness across many health dimensions [e.g. physical, spiritual, psychological, social, emotional, intellectual (Adams, Bezner, & Steinhardt, 1997), and across many functional aspects of well-being [e.g. creative, coping, social, essential, physical (Hattie, Myers, & Sweeney, 2004). They include positive and negative mood states [e.g. happy, lively, annoyed (Curran, Andrykowski, & Studts, 1995; Thompson, 2007; Watson & Clark, 1999), attitudinal aspects of well-being [e.g. optimistic about the future, feel full of energy (Bishop & Yardley, 2010; Herzberg, Glaesmer, & Hoyer, 2006), positive affect [Patient-Reported Outcome Measurement Information System (PROMIS) item banks (Salsman et al., 2020), life purpose, optimism, sense of coherence, perceived wellness (Adams et al., 1997; Adams, Bezner, Drabbs, Zambarano, & Steinhardt, 2000; Adams, Bezner, Garner, & Woodruff, 1998), mental health (Hays, Bjorner, Revicki, Spritzer, & Cella, 2009), and dysphoria/apathy (e.g. little interest or pleasure in doing things, feeling hopeless) (Smith, Gotman, Lin, & Yonkers, 2010; Thombs et al., 2014). These conceptualizations of wellness can be used as a basis for counseling interventions (Myers, Sweeney, & Witmer, 2000; Roscoe, 2009). Despite these many commonly used measurement tools, we believe that they tend to miss what we believe we have shown to be authentic aspects of wellness.

Accordingly, the present work sought to validate a psychometric measure based on this expanded conceptualization of wellness, using approaches from both modern and classical test theory.

## Materials and methods

### Design

This cross-sectional study was administered in late Spring through mid-Summer of 2020, as part of a larger longitudinal study of the impact of the COVID-19 pandemic on health and well-being.

### Sample and procedure

This study recruited participants via Rare Patient Voice (RPV) and Ipsos-Insight (IPSOS), the former to target patients and caregivers of patients with chronic medical conditions (the sicker group), and the latter to target a comparison sample of United States (US) adults. RPV is a panel-research organization that recruits participants with rare and not-so-rare chronic illnesses and their caregivers. By attending patient-advocacy and professional medical conferences that welcome patients and family members, RPV is able to vet the participants as truly having the condition they claim. RPV retains and grows its participant base by providing a range of incentives for study participation. For funded research, RPV has an honorarium structure that links amount of payment to time required to complete the survey. For unfunded academic research (such as the present work), RPV seeks to ensure that the study objectives are likely to be personally meaningful to the study participants and that the investigators will provide lay-language summaries of study findings. Ipsos-Insight is a global market research company that facilitates access to nationally representative samples. Our Ipsos-Insight sample was selected to be representative of the general United States population in terms of age, gender, region, and income distributions (the healthier group). Their study participants are compensated with an Ipsos-Insight point system. Eligible participants were age 18 or older and able to complete an online questionnaire. This survey was administered through the Health Insurance Portability and Accountability Act (HIPAA)-compliant, secure Alchemer engine (www.alchemer.com). The protocol was reviewed and approved by the New England Independent Review Board (NEIRB #2021164), and all participants provided informed consent prior to beginning the survey.

### Measures

The study included the *DeltaQuest Wellness Measure©* (DQ Wellness) as well as six widely used person-reported QOL outcomes for construct-validity comparisons.

DQ Wellness is a 15-item measure tapping attitudinal, cognitive, and behavioral aspects of wellness. Two of the three authors of the present manuscript (CES and RBS) wrote the 15 items based on the conceptual model (Figure 1). The idea of this conceptual model is that these various aspects reflect an underlying and overall concept of wellness. We then invited feedback on item clarity and face validity from experienced, measurement-development colleagues and clinicians working with a range of medical populations. Thirteen positively-worded items assessed concepts such as joy, zest, self-care, calm, and a positive engagement in the world and with others. Two negatively-worded items tapped characteristics believed antithetical to wellness, namely low energy and preoccupation with the negative aspects of one’s life. These negatively-worded items were reverse-coded for scoring so that higher scores on the DQ Wellness reflect higher wellness. All items followed an instruction to ‘indicate how true each of the following statements is for you over the past week’ and used rating-scale descriptors ranging from ‘not at all’ (0) to ‘very much’ (4). All items provided an option ‘do not know/prefer not to answer,’ which, if used, was coded as missing (−99).

The QOL measures included for construct-validation purposes were: (1) the *Patient-Reported Outcome Measurement Information System* (*PROMIS)−10* measure of general physical and mental health; 10 items (Hays et al., 2009); (2) the *NeuroQOL Applied Cognition* (Cella et al., 2012) measures of perceived difficulties in everyday cognitive abilities (memory, attention, and decision-making; 8 items) and applications of mental function (planning, organizing, calculating, working with memory and learning; 8 items); (3) the *NeuroQOL Positive Affect and Well-Being* 9-item short-form (Cella et al., 2012); and (4) the *Purpose in Life* and (5) *Environmental Mastery* 7-item subscales of the Ryff Psychological Well-Being Scale (Ryff, 1989). These six subscales have strong documented reliability and validity (Cella et al., 2012; Ryff, 2014). High scores indicate better outcomes for PROMIS Physical and Mental, NeuroQOL Positive Affect and Wel-Being, and Ryff Purpose in Life and Environmental Mastery, and worse outcomes for NeuroQOL Applied Cognition.

### Demographic characteristics

Data collected included year of birth, gender, cohabitation/marital status, employment status, ethnicity, race, education, height, weight, difficulty paying bills, with whom the person lives, smoking status, year of chronic medical diagnosis (if applicable), and whether receiving help to complete survey.

### Analysis

Psychometric analysis used approaches from item response theory (IRT) and classical test theory. The sample was randomly divided into training and validation subsamples, each comprising 50% of the sample. This step, a simple form of cross-validation, enables one to assess how generalizable results are across sample subgroups. Final parameter estimates were then based on the full sample. Exploratory factor analysis (EFA) was performed using Mplus (Muthén & Muthén, 1998–2019) on the training sample followed by confirmatory factor analysis (CFA) in the validation sample. We began with EFA with Geomin (oblique) rotation, evaluating one-, two-, three-, and four-factor solutions to describe our 15-item measure. The two negatively-worded items were reverse-coded prior to CFA. We implemented CFA, testing first a one-factor model with four and five residual correlations, then a bifactor model with one general factor and four specific factors. The bifactor model is a useful tool for exploring dimensionality, particularly in the context of a conceptually broad construct (Reise, Morizot, & Hays, 2007). The bifactor model enables one to investigate multidimensional constructs that comprise different dimensions but share a common, general factor (Carona, Moreira, Halberstadt, & Fonseca, 2021; Reise et al., 2007). These specific subfactors account for the unique influence of the specific sub-dimensions, over and above the general factor (Chen, West, & Sousa, 2006). The bifactor model has proven useful for the development of short-form measures that tap a number of sub-constructs which all relate to a general construct (e.g. Carona et al., 2021; De Bruin & Du Plessis, 2015; Hides et al., 2016; Jovanović, 2015; Levant, Hall, & Rankin, 2013; Levant, Hall, Weigold, & McCurdy, 2016; McDermott, Levant, Hammer, Borgogna, & McKelvey, 2019; Neff, Tóth-Király, Knox, Kuchar, & Davidson, 2021). The bifactor model is distinct from a hierarchical factor model where a single higher-order factor gives rise to some number of lower-order factors. Instead, in the bifactor model, the specific factors are extracted so as to be uncorrelated with each other and with the general factor (Edwards, Wirth, Houts, & Bodine, 2014). As is customary with bifactor models, incorporating the residual correlation into the general score enables the creation of short-form measures that represent multiple sub-domains within a broader construct (Chen, Hayes, Carver, Laurenceau, & Zhang, 2012; Reise et al., 2007). As a last step, we repeated the bifactor model as a two-group model to compare factor means between the sicker and healthier groups.

The final model structure was evaluated for measurement invariance between male and female respondents. All CFA analysis used weighted least squares mean- and variance-adjusted (WLSMV) estimation, and it used as its default listwise deletion (Muthén & Muthén, 1998–2019). Model fit focused on the Root Mean Square Error of Approximation (RMSEA) and the Comparative Fit Index (CFI) using standard criteria for good fit [i.e. RMSEA <0.08, CFI ≥0.90 (Hu & Bentler, 1999). We evaluated whether a short list of items performed better than the full set (Stucky, Thissen, & Orlando Edelen, 2013). IRT analyses then built on this final bifactor model using a Graded IRT model (Samejima, 2016) to examine item characteristics, to identify poorly functioning items, and to compute slopes, intercepts, and thresholds. This model also computed item information functions, item trace lines, and the marginal reliability^1^ of the scaled scores, and it enabled the creation of an IRT scoring table based on the summed score. The IRT analysis used marginal maximum likelihood and so all response patterns were analyzed whether data were missing or otherwise.

Construct validity was assessed in four ways. First, we hypothesized that the DQ Wellness general score would be responsive to increases in comorbidities (general construct validity). Response distributions were compared across levels of reported comorbidities using a paneled histogram. Second, Pearson correlation coefficients between the DQ Wellness general score and the established PROs were used to test construct-validity hypotheses. We hypothesized the following convergent-divergent validity suppositions: the DQ Wellness general score would be highly correlated with the other well-being measures (Positive Affect & Wellbeing, Purpose in Life, and Environmental Mastery), but not so highly correlated as to indicate complete overlap of the latent trait (0.7< |r| <0.9). If there were complete overlap, there would be no need for a new measure of wellness. We hypothesized that the DQ Wellness general score would be moderately correlated with global mental health (0.45< |r| <0.65), because wellness is part of mental health and vice versa. We hypothesized that the DQ Wellness general score would be moderately but less highly correlated with global physical health and applied cognition (0.3< |r| <0.45). A third type of construct validity test compared known groups on the standardized factor means for the DQ Wellness general score. We expected the sicker group to score lower than the healthier group on the DQ Wellness general score. Finally, a multiple regression model included as independent variables the five measures related to construct-validity testing, and DQ Wellness general score as a dependent variable. This model enabled computing how much these other measures explained variance in DQ Wellness general score (i.e. unique variance), and comparing the relative importance of each measure to the DQ Wellness general score.

Measurement invariance by gender was assessed across the DQ Wellness general score’s factor loadings (i.e. metric invariance) and item thresholds (i.e. scalar invariance) using the chi-square difference testing in Mplus. Because the bifactor models were fit using WLSMV estimation, the DIFFTEST option was implemented to provide a corrected chi-square across nested models. The approach to assessing invariance across both loadings and thresholds began by fitting a baseline model with fixed factor loadings and thresholds across males and females, with the factor means of the female group allowed to vary and account for potential mean gender differences. Next, in an iterative manner across each item, the general factor loadings and thresholds were freed, resulting in 30 chi-square tests (15 general factor loadings and 15 item thresholds). Any potential bias in relationship between each DQ Wellness item and gender, above and beyond simple factor mean differences, would be captured by the subsequent chi-square tests.

Statistical analyses were implemented using IBM SPSS version 26 (IBM, 2019), Mplus version 8.4 (Muthén & Muthén, 1998–2015), and IRT PRO version 3.1 (Cai, Du Toit, & Thissen, 2011–2015).

## Results

### Sample

The study sample included 4,816 persons: 3,085 RPV patients, 685 RPV caregivers, 191 RPV participants who are both patients and caregivers, and 855 in the IPSOS comparison group. The sample was heterogeneous across age, gender, socioeconomic status, health status, and US geographic region. Table 1 provides sociodemographic characteristics and the most prevalent illnesses, and Table 2 provides the descriptive statistics of the patient-reported outcomes (PROs) used for construct validity. The sample had a mean age of 51.6 (standard deviation [SD] = 14.2), and 82% were female. The sample was 83% white (5% Hispanic) and 6% black. Sixty-two percent of respondents were married or in a domestic partnership, and 12% were living alone. Although self-identified as ‘caregivers’ in the RPV sample, these individuals reported almost as many comorbidities as the ‘patients’ (3.3 vs. 4.0) (Table 1). Similarly, the IPSOS comparison sample reported an average of 2.5 comorbidities (Table 1). Thus, all study participants were dealing with some degree of health challenges, reflecting the abovementioned labels of ‘sicker’ (RPV) and ‘healthier’ (IPSOS). Supplemental Table 1 provides the descriptive characteristics separately for the ‘sicker’ and ‘healthier’ subgroups.

**Table 1.** Sample demographic characteristics (N = 4,816).

Variable		#	%
Role	Patient	3085	64%
	Caregiver	685	14%
	Both	191	4%
	Comparison Sample	855	18%
Age	Mean (SD)	51.6	14.2
Gender	Male	857	18%
	Female	3930	82%
	Other	23	0.5%
	Missing	6	0.1%
Living Alone		584	12%
Marital Status	Never Married	779	16%
	Married	2675	56%
	Cohabitation/ Domestic Partnership	328	7%
	Separated	91	2%
	Divorced	677	14%
	Widowed	242	5%
	Missing	24	0.5%
Ethnicity	Hispanic or Latino	225	5%
	Missing	130	3%
Race	Black or African American	308	6%
	White	4291	89%
	Other	215	4%
	Missing	2	0%
Country Mother Born	United States	4351	90%
	Canada	98	2%
	United Kingdom	47	1%
	Germany	31	1%
	Others	287	6%
	Missing	2	0.0%
Country Father Born	United States	4305	89%
	Canada	88	2%
	United Kingdom	40	1%
	Mexico	40	1%
	Others	341	7%
	Missing	2	0.0%
Difficulty Paying Bills	Not at all Difficult	2312	48%
	Slightly Difficult	1089	23%
	Moderately Difficult	708	15%
	Very Difficult	325	7%
	Extremely Difficult	268	6%
	Missing	114	2%
Employment Status	Employed	1976	41%
	Unemployed	603	13%
	Retired	920	19%
	Disabled Due To Medical Condition	1244	26%
	Missing	73	2%
Education	Less than high school graduate	56	1%
	High school diploma/GED	463	10%
	Trade or technical degree	314	7%
	Some college	1309	27%
	College degree	1420	29%
	Postgraduate degree	1237	26%
	Missing	17	0.4%
BMI	Mean (SD)	30	8.3
Currently Smoke or Vape	Not at all	3982	83%
	Some days	248	5%
	Every day	548	11%
	Missing	38	1%
Comorbidities (of 15 presented)			
Patients	Mean (SD)	4	2.3
Caregivers (incl. Patient-Caregivers)	Mean (SD)	3.3	2.4
Comparison Sample	Mean (SD)	2.5	2.8
All	0	269	6%
	1	629	13%
	2	802	17%
	3	829	17%
	4	735	15%
	5	596	12%
	6	417	9%
	7 or more	528	11%
	Missing	11	0.2%
Time Since Diagnosis (if applicable)	Mean no. years (SD)	14.9	14.1
Disease Category	Less Common Cancers	933	19%
	Multiple Sclerosis	607	13%
	Common Cancers, Not Breast	214	4%
	Breast Cancer	169	4%
	Autoimmune	26	1%
Received Help Completing Survey	Yes	78	2%

Some sets of percentages may not add up to 100% due to rounding.

GED = General Educational Development (i.e. high-school equivalency test)

SD = standard deviation

**Table 2.** Descriptive statistics of person-reported outcomes used for construct validity.

								95% CI of Pearson r	
	N	Min	Max	Mean	Std. Deviation	Skewness	Pearson r with DQ Wellness	Lower Limit	Upper Limit
DeltaQuest WellBeing	4792	−3.44	2.36	−0.27	0.91	0.03	−	−	−
PROMIS-10 Global Physical Health (T score)	4810	16.2	67.7	41.77	9.81	0.17	0.53	0.51	0.55
PROMIS-10 Global Mental Health (T score)	4806	21.2	67.6	44.32	9.64	0.08	0.75	0.73	0.76
NeuroQOL Applied Cognition (low = better)	4808	16	80	31.92	14.41	0.93	−0.49	−0.51	−0.47
NeuroQOL Positive Affect & Well-Being	4729	26.3	68	51.36	7.71	0.06	0.83	0.82	0.84
Ryff Purpose in Life	4803	7	42	29.58	6.87	−0.31	0.62	0.60	0.64
Ryff Environmental Mastery	4800	5	42	28.47	8.21	−0.24	0.74	0.73	0.75

### Psychometric results

#### Factor analyses

Supplemental Table 2 provides item descriptive statistics and Supplemental Figure 1 shows item histograms. Items reflected endorsement at all response options and generally had normal distributions and low skewness statistics. In the EFA, one-, two-, three-, and four-factor models with Geomin (oblique) rotation failed to produce simple structure. Supplemental Table 3 shows results of these EFA models, and Supplemental Figure 2 shows a scree plot. The scree plot indeed suggests that a second factor explains substantially less variance than the first but had an eigenvalue greater than 1.0. The three- and four-factor EFAs had good model-fit statistics but did not yield four three or four interpretable factors, respectively. This content-based information in addition to the scree plot supported the idea of a bifactor model, i.e. the existence of a general factor with residual correlations among several sets of items.

**Figure 2.**
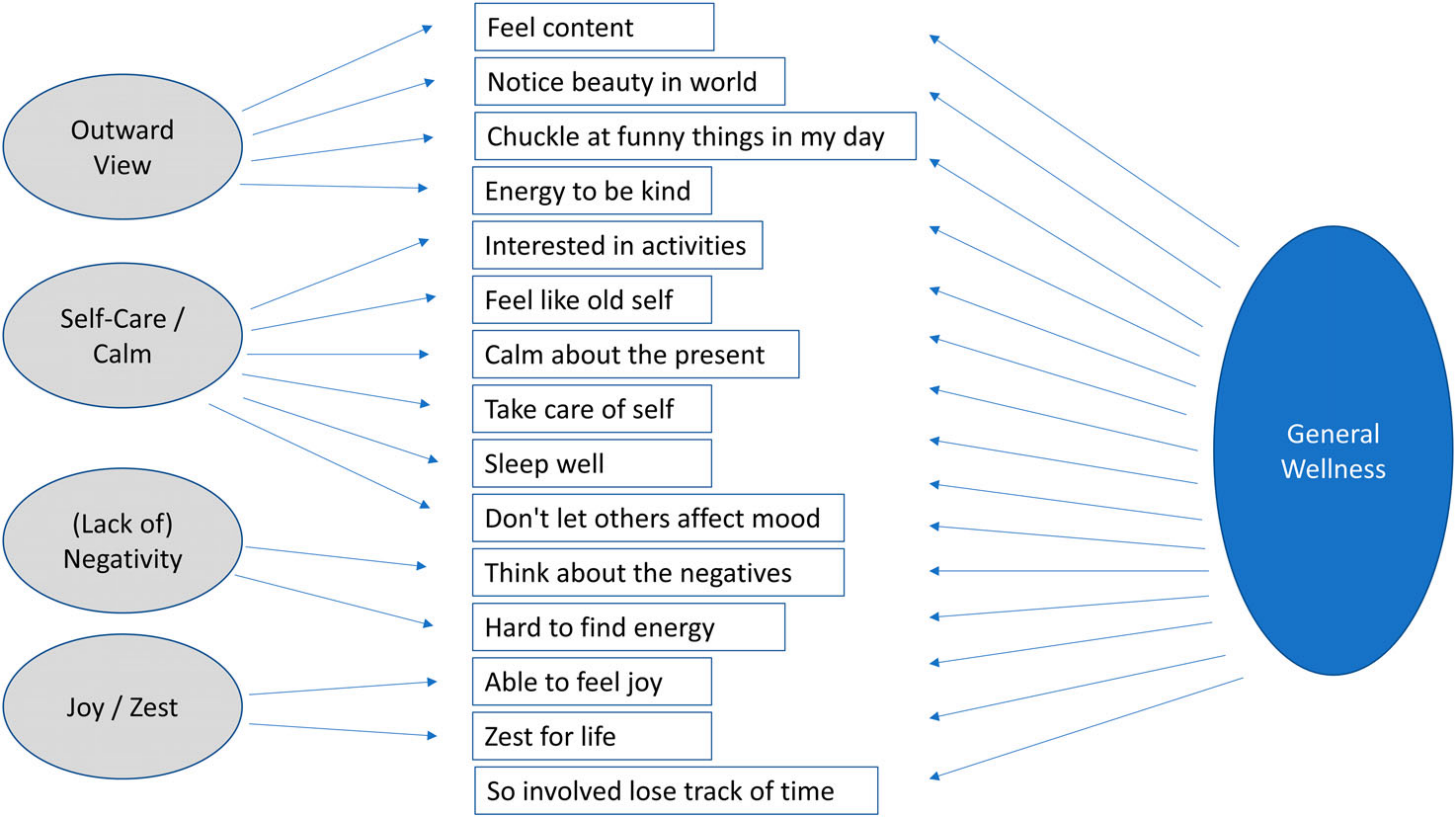
Bifactor Model of Wellness. This bifactor model comprises one general factor and four specific factors. Incorporating the specific factors’ residual correlations into the general score enables the creation of a short-form measure that represents multiple sub-domains within a broader construct.

A CFA testing a one-factor model with four and five residual correlations did not fit the data well (RMSEA = 0.87 and 0.85, CFI = 0.974 and 0.975, TLI = 0.968 and 0.970, respectively). We thus tested a bifactor model with four specific factors to account for residual correlations. All models were tested first in a training subsample (50% of sample) and confirmed in a validation subsample (the remaining 50%). Table 3 shows the final two-group bifactor solution on the full sample (n = 4,816). This bifactor model fit the data well (RMSEA = 0.063, CFI = 0.982, TLI = 0.983). It showed that all items loaded highly on the first factor, which we call ‘DQ Wellness general score.’ In addition to this overall factor, there were four specific factors reflecting what we labeled Outward View, Self-Care/Calm, (Lack of) Negativity, and Joy / Zest (see Figure 2). All in all, 58% of the variance in the 15 items was explained by the bifactor model. Of the whole, 49.0% was explained by DQ Wellness general score, and 3.5%, 2.9%, 1.9%, and 0.7% were explained by the Outward View, (Lack of) Negativity, Self-Care/Calm, and Joy / Zest specific factors, respectively.

**Table 3.** Confirmatory Factor Analysis: Two-group Bifactor Model Loadings (n = 4,815).

Item	Wellness	Outward View	Self-Care / Calm	(Lack of) Negativity	Joy / Zest
Feel content	0.91	0.08			
Notice beauty in world	0.77	0.46			
Chuckle at funny things in my day	0.74	0.24			
Energy to be kind	0.73	0.54			
Interested in activities	0.84		0.10		
Feel like old self	0.82		0.26		
Calm about the present	0.80		0.19		
Take care of self	0.74		0.15		
Sleep well	0.68		0.31		
Don't let others affect mood	0.64		0.19		
Think about the negatives*	0.35			0.29	
Hard to find energy*	0.31			0.29	
Able to feel joy	0.89				0.24
Zest for life	0.86				0.24
So involved lose track of time	0.35				

* Note: These negatively-worded items are reverse-coded for creating the Wellness score.

The singular DQ Wellness general score can be used to summarize the individual’s wellness. Because the factor loadings and explained variance for the specific factors were generally low, whatever variance was explained was largely accounted for by the general factor. The specific factors’ low factor loadings also indicate that their scores would have lower reliability than generally considered acceptable, so they were not considered in subsequent analyses. Supplemental Table 4 shows the marginal slopes, intercepts, and thresholds for the bifactor model. Supplemental Figure 3 provides the item information functions and item characteristic curves for the DQ Wellness. This figure suggests that five items provide particularly good information about the latent trait (content, joy, zest, interested, old self). Future end users can generate scores using this same total. Accordingly, one will be able to compare scores across other samples.

#### Reliability

**Figure 3.**
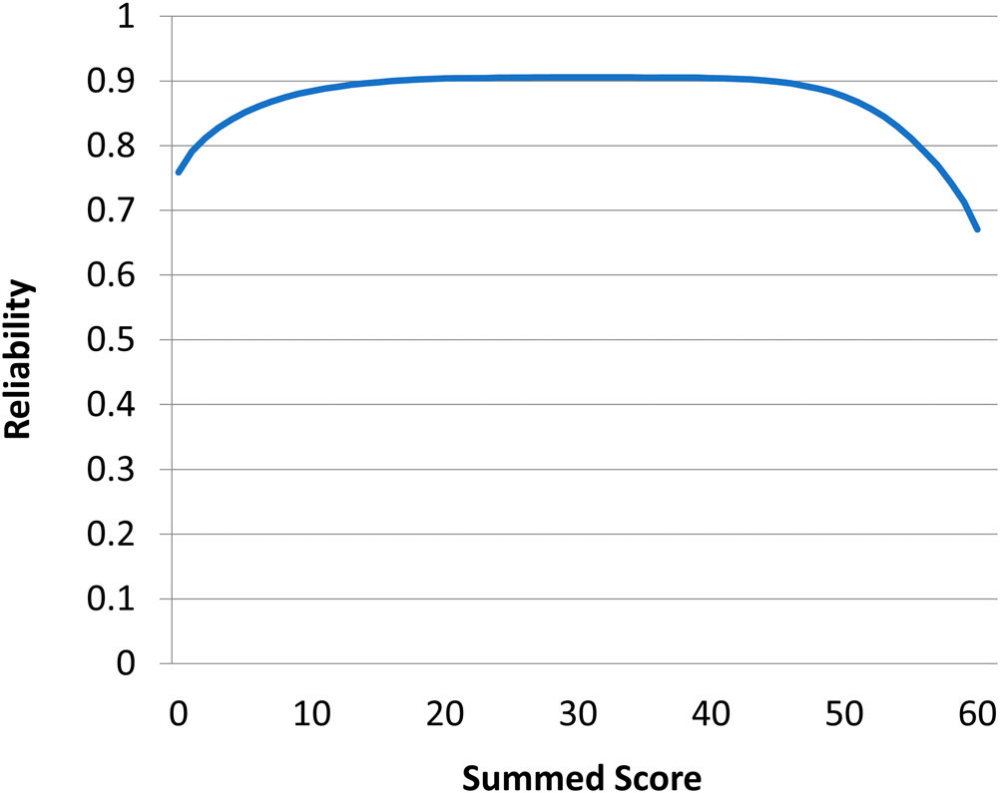
Score Reliability Across the DQ Wellness General Score. The DQ Wellness score exhibits high reliability across all levels of the summed score, and thus, across the full range of the latent trait of Wellness.

Based on the IRT parameterization of the bifactor model, the marginal reliability of the DQ Wellness general score was 0.89, and the measure’s score reliability was uniformly high across nearly all levels of wellness (Figure 3).

#### Construct validity

**Figure 4.**
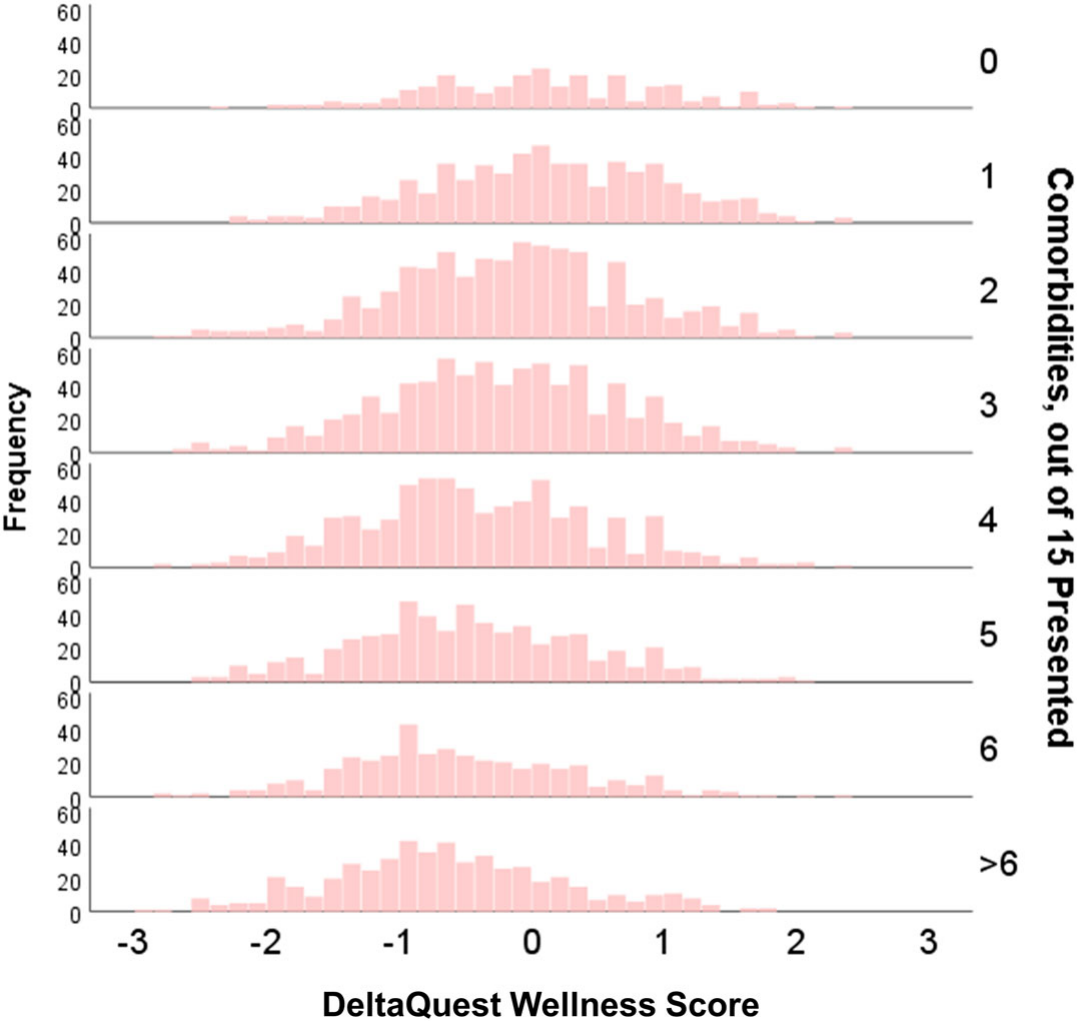
Paneled Histogram of the DQ Wellness General Score by Comorbidity Burden. As numbers of comorbidities increase, the mean and distribution of the DQ Wellness general score shift lower.

DQ Wellness general score demonstrated good general construct validity when considered in connection with number of comorbidities. Figure 4 shows a paneled histogram displaying a notable and steady reduction in DQ Wellness general score occurring at each additional comorbidity.

Convergent and divergent validity were demonstrated using Pearson correlation coefficients between the DQ Wellness general score and the PROs used to establish construct validity (Table 2). As hypothesized, DQ Wellness general score was highly and positively correlated with the other well-being measures but not so highly correlated as to suggest that they all measure the same latent trait (0.49<|r|<0.83). Also as expected, the DQ Wellness general score was moderately correlated with better mental health (r = 0.75). It was less highly correlated with better physical health (r = 0.53) and better applied cognition (r = −0.49), although it had a slightly higher-than-hypothesized correlation with the latter (hypothesis r<|0.45|).

In addition to these tests of convergent and divergent validity, construct validity was examined via known-groups validity. The sick and healthy groups had DQ Wellness means (with SDs) of −0.30 (0.90) and −0.01 (0.98), respectively. The standardized factor means for the sicker and healthier groups are reported in SD to reflect effect sizes. In the context of the bifactor model which constrained the slopes and thresholds to be equal across groups, the sicker population scored on average 0.25 SD below the healthier population on DQ Wellness general score.

From the multiple regression model, 23% of the variance in the DQ Wellness general score was not explained by the other measures of QOL and well-being (i.e. tolerance = 0.23, Table 4). Notably, even among the three established PROs that were the best predictors of the DQ Wellness general score (NeuroQOL Positive Affect & Well-Being, PROMIS Mental, Ryff Environmental Mastery), tolerance was as low as 0.33–0.39. These findings support the idea that the DQ Wellness is distinct from other measures related to well-being.

**Table 4.** Multivariate Regression Model Predicting General Wellness Score (Adjusted R^2^ = 77.2%).

	Standardized Coefficients			Collinearity Statistics	
	Beta	t	Sig.	Tolerance	VIF
Ryff Environmental Mastery	0.186	15.39	0.000	0.33	3.00
Ryff Purpose in Life	0.028	2.82	0.005	0.49	2.04
PROMIS-10 Physical (T-score)	0.084	9.52	0.000	0.63	1.59
PROMIS-10 Mental (T-score)	0.192	16.81	0.000	0.37	2.68
NeuroQOL Applied Cognition	−0.029	−3.21	0.001	0.59	1.69
NeuroQOL Positive Affect & Well-Being	0.500	44.73	0.000	0.39	2.57

#### Measurement invariance

The males and females had DQ Wellness means (with SDs) of −0.11 (0.96) and −0.31 (0.90), respectively. Results from measurement invariance testing indicated only modest differences in factor loadings by gender (Table 5). Across the 15 factor-loading-invariance tests, three indicated statistically significant effects (*p* < .05) involving three items, and one of these items also had statistically-significant threshold invariance. The two reverse-scored items had slight general factor loading differences: Item 3 (‘ … Hard to find energy’) had a more positive loading for men than women (0.08 vs −0.35, respectively) and item 6 (‘ … think about the negatives’), the reverse (−0.35 vs −0.07, respectively). Item 15 (‘ … So involved I lose track of time’) was more related to the DQ Wellness general score for men than women (loadings of 0.76 vs. 0.37). Threshold differences were detected for this latter item, indicating that men were more likely to endorse this item after controlling for overall gender differences in DQ Wellness general score (male thresholds: *b*_1_ = −1.55, *b*_2_ = −0.71, *b*_3_ = 0.06, *b*_4_ = 0.83; female thresholds: *b*_1_ = −1.11, *b*_2_ = −0.45, *b*_3_ = 0.36, *b*_4_ = 1.28). Even so, given that the measurement invariance identified few significant effects, and that those that were detected involved items with lower factor loadings that have only a minor impact on the DQ Wellness general score, we recommend retaining all 15 items.

**Table 5.** Results of measurement invariance analyses by gender.

	General Factor Loading Measurement Invariance			Threshold Measurement Invariance		
Item Label	Chi-square value	df	*p*-value	Chi-square value	df	*p*-value
Interested in activities	0.09	1	0.76	6.97	4	0.14
Feel like old self	0.04	1	0.84	3.88	4	0.42
Hard to find energy	**32**.**29**	1	**0**.**00**	8.37	4	0.08
Zest for life	0.01	1	0.92	6.18	4	0.19
Able to feel joy	0.25	1	0.62	6.84	4	0.14
Think about the negatives	**12**.**38**	1	**0**.**00**	8.39	4	0.08
Calm about present	0.58	1	0.45	2.39	4	0.66
Sleep well	1.80	1	0.18	4.59	4	0.33
Chuckle at funny things in my day	1.87	1	0.17	4.88	4	0.30
Feel content	0.31	1	0.58	4.50	4	0.34
Take care of self	0.00	1	0.98	3.77	4	0.44
Don't let others affect me	0.83	1	0.36	1.31	4	0.86
Notice the beauty in world	0.51	1	0.48	3.88	4	0.42
Energy to be kind	0.06	1	0.81	3.71	4	0.45
So involved lose track of time	**13**.**53**	1	**0**.**00**	**19**.**92**	**4**	**0**.**00**

## Discussion

The present study supports the cross-sectional reliability and construct validity of the DQ Wellness measure. IRT analyses supported a bifactor structure of the measure, such that one DQ Wellness general score can be used to summarize the individual’s wellness. The bifactor model not only fit the data, but the idea of specific factors is consistent with the conceptual model. This DQ Wellness general score is computed using an IRT scoring table. The score demonstrated all four tested aspects of construct validity and showed only modest gender effects on measurement invariance.

The measure taps aspects of wellness that are not included in other commonly used measures of wellness or well-being, aspects that research has shown to be relevant and important. While wellness is related to physical health, it is not the same as physical health (i.e. a correlation of 0.53 suggests that higher levels of physical health are associated with higher levels of wellness, but they are not the same construct). Further, the small difference (0.25 SD) in factor means between the sicker and healthier groups seems consistent with the idea that wellness only partially reflects physical health.

To capture all six sub-constructs originally envisioned for the DQ Wellness measure (Figure 1), one could patch together existing measures for each, resulting in an instrument of over 100 items. We have created a psychometrically sound, brief measure with only 15 items. We hope that this measure will serve important purposes in observational and interventional research as well as for clinical interventions such as mindfulness, coping, or rehabilitation.

The present study represents an initial validation of the DQ Wellness measure. As with any measure, the validation process is iterative. In this first pass, we have established that the measure has good psychometric properties, including important aspects of construct validity and internal consistency reliability. It measures a construct distinct from simply ‘good health,’ and more than mental health and eudaemonic well-being. Although three items had relatively low DQ Wellness general score factor loadings, they were retained in light of the need to balance homogeneity and reliability with domain coverage and content validity (Dawis, 2000). One of the items addresses avoiding being caught up in negativity; one addresses vitality; and one addresses the concept of flow (Csikszentmihalyi, Abuhamdeh, & Nakamura, 2014; Nakamura & Csikszentmihalyi, 2014). Their content helps to round out the scale as we envisioned it in the conceptual model (Figure 1). It is possible that future research with other patient and non-patient populations may further support the added value of these three items. Accordingly, they are retained in the measure on this basis of this initial validation study. Future research might also develop the measure further by comparing it to other potential operationalizations of wellness. For example, the Caprara et al. Positivity Scale assesses the tendency to view life and experiences with a positive outlook (Caprara et al., 2012). The Dambrun Selflessness scale measures self-transcendence and connectedness to others (Dambrun, 2017). Maslow’s concept of self-actualization might also be related to DQ Wellness general score, and has been operationalized with a number of measures (Jones & Crandall, 1986; Lefrancois, Leclerc, Dubé, Hebert, & Gaulin, 1997; Shostrom, 1964; Sumerlin & Bundrick, 1996). The Rogerian concepts of genuineness, acceptance and empathy (Rogers, 1965) are also likely related to DQ Wellness general score, as they have been found to be core components of personal growth during adversity as well as of effective therapeutic relationships (Kirschenbaum & Jourdan, 2005). Indeed, wellness is likely a complex and multi-faceted construct, so any brief measure may miss important aspects in the service of pragmatism. We acknowledge that the best conceptualization of wellness may comprise facets including diet, exercise, certain specific aspects of social interaction, spirituality, emotional intelligence, or incorporation of medical knowledge, to name a few. Nonetheless, we believe that the DQ Wellness measure captures important facets more broadly than other current tools, supporting its use in future research.

While this study has clear advantages in terms of notable sample size and the inclusion of a general-population comparison group, its limitations should be noted. First, it is only able to address the reliability and validity of the DQ Wellness measure cross-sectionally. Future research will need to address the longitudinal construct validity, including the measure’s responsiveness to change in circumstances, and scores’ stability in the absence of such change. Future research might also address extrinsic convergent validity (Gonzalez, MacKinnon, & Muniz, 2021) by comparing DQ Wellness’ correlations with external criteria to those found for other measures of wellness and well-being. Additionally, the sample over-represented females, people of Caucasian race, and people with chronic illness. Future research should assess the generalizability of study findings to samples with more even distributions of gender, race, and health status. Also, the current data collection took place during the COVID-19 global pandemic, which may have altered relationships among the items and other study variables. It would be worthwhile to revisit the measure’s psychometric characteristics when these hopefully anomalous times are behind us.

In summary, we present a new, relatively brief measure of a construct that could be useful for observational or interventional studies, that taps content not adequately assessed by other measures. Interested potential users may contact the first author for access to the measure and scoring protocol.

## Supplementary Material

Supplemental Material

## Data Availability

The study data are confidential and thus not able to be shared.

## Note

1 The marginal reliability coefficient is the proportion of variance in the observed score due to true score (Reise & Revicki, 2014). It may be used for comparison with classical reliability statistics (Reise & Revicki, 2014). A generally accepted rule is that α of 0.6-0.7 indicates an acceptable level of reliability, and 0.8 or greater a very good level (Reeve et al., 2013).

## Materials and/or code availability

Requests for software code will be considered, and will be made available if deemed reasonable.
