# Supplementary material for: Expanding the purview of wellness indicators: validating a new measure that includes attitudes, behaviors, and perspectives
Source: Health Psychol Behav Med. 2021 Dec 1;9(1):1031–52. doi: 10.1080/21642850.2021.2008940 (PMC8648008; doi:10.1080/21642850.2021.2008940)
Supplement: Supplemental Material [file RHPB_A_2008940_SM8555.zip › DQ Wellness_Supp Table 1.docx]

| **Table 1. Demographic Characteristics by Sick vs. Well** (N=4,816) | | | | | | |
| --- | --- | --- | --- | --- | --- | --- |
|  |  |  | **Sick** (N = 4,316) | | **Well** (N = 500) | |
| **Variable** |  |  | **#** | **%** | **#** | **%** |
| Role |  | Patient | 3084 | 71% | 1 | 0% |
|  |  | Caregiver | 683 | 16% | 2 | 0% |
|  |  | Both | 191 | 4% | 0 | 0% |
|  |  | Neither | 358 | 8% | 497 | 99% |
| Gender |  | Male | 612 | 14% | 245 | 49% |
|  |  | Female | 3677 | 85% | 253 | 51% |
|  |  | Other | 21 | 0% | 0 | 0% |
|  |  | Missing | 6 | 0% | 2 | 0% |
| Living Alone |  | Yes | 480 | 11% | 104 | 21% |
| Marital Status |  | Never Married | 667 | 15% | 112 | 22% |
|  |  | Married | 2422 | 56% | 253 | 51% |
|  |  | Cohabitation/ Domestic Partnership | 306 | 7% | 22 | 4% |
|  |  | Separated | 86 | 2% | 5 | 1% |
|  |  | Divorced | 622 | 14% | 55 | 11% |
|  |  | Widowed | 190 | 4% | 52 | 10% |
|  |  | Missing | 23 | 1% | 1 | 0% |
| Ethnicity |  | Hispanic or Latino | 192 | 4% | 33 | 7% |
|  |  | Missing | 122 | 3% | 8 | 2% |
| Race |  | Black or African American | 270 | 6% | 38 | 4% |
|  |  | White | 3879 | 90% | 412 | 43% |
|  |  | Other | 165 | 4% | 500 | 53% |
|  |  | Missing | 2 | 0% | 0 | 0% |
| Country Mother Born |  | United States | 3922 | 91% | 429 | 86% |
|  |  | Canada | 94 | 2% | 4 | 1% |
|  |  | United Kingdom | 43 | 1% | 4 | 1% |
|  |  | Germany | 30 | 1% | 1 | 0% |
|  |  | Others | 225 | 5% | 62 | 12% |
|  |  | Missing | 2 | 0% | 0 | 0% |
| Country Father Born |  | United States | 3886 | 90% | 419 | 84% |
|  |  | Canada | 85 | 2% | 3 | 1% |
|  |  | United Kingdom | 35 | 1% | 5 | 1% |
|  |  | Mexico | 37 | 1% | 3 | 1% |
|  |  | Others | 271 | 6% | 70 | 14% |
|  |  | Missing | 2 | 0% | 0 | 0% |
| Difficulty Paying Bills |  | Not at all Difficult | 2054 | 49% | 258 | 52% |
|  |  | Slightly Difficult | 1010 | 24% | 79 | 16% |
|  |  | Moderately Difficult | 634 | 15% | 74 | 15% |
|  |  | Very Difficult | 290 | 7% | 35 | 7% |
|  |  | Extremely Difficult | 229 | 5% | 39 | 8% |
|  |  | Missing | 9 | 0% | 15 | 3% |
| Employment Status |  | Employed | 1755 | 41% | 221 | 44% |
|  |  | Unemployed | 526 | 12% | 77 | 15% |
|  |  | Retired | 756 | 18% | 164 | 33% |
|  |  | Disabled Due To Medical Condition | 1210 | 28% | 34 | 7% |
|  |  | Missing | 69 | 2% | 4 | 1% |
| Education |  | Less than high school graduate | 45 | 1% | 11 | 2% |
|  |  | High school diploma/GED | 387 | 9% | 76 | 15% |
|  |  | Trade or technical degree | 287 | 7% | 27 | 5% |
|  |  | Some college | 1177 | 27% | 132 | 26% |
|  |  | College degree | 1268 | 29% | 152 | 30% |
|  |  | Postgraduate degree | 1135 | 26% | 102 | 20% |
|  |  | Missing | 17 | 0% | 0 | 0% |
| Currently Smoke or Vape |  | Not at all | 3632 | 84% | 350 | 70% |
|  |  | Some days | 191 | 4% | 57 | 11% |
|  |  | Every day | 460 | 11% | 88 | 18% |
|  |  | Missing | 33 | 1% | 5 | 1% |
| Comorbidities |  | 0 | 154 | 4% | 115 | 23% |
|  |  | 1 | 519 | 12% | 110 | 22% |
|  |  | 2 | 726 | 17% | 76 | 15% |
|  |  | 3 | 751 | 17% | 78 | 16% |
|  |  | 4 | 691 | 16% | 44 | 9% |
|  |  | 5 | 572 | 13% | 24 | 5% |
|  |  | 6 | 402 | 9% | 15 | 3% |
|  |  | 7 or more | 492 | 11% | 36 | 7% |
|  |  | Missing | 9 | 0% | 2 | 0% |
| Disease Category |  | Less Common Cancers | 874 | 20% | 57 | 12% |
|  |  | Multiple Sclerosis | 607 | 14% | 0 | 0% |
|  |  | Common Cancers, Not Breast | 214 | 5% | 0 | 0% |
|  |  | Breast Cancer | 169 | 4% | 0 | 0% |
|  |  | Autoimmune | 26 | 1% | 0 | 0% |
| Received Help Completing Survey |  | Yes | 5 | 0% | 19 | 4% |
|  |  |  | **Mean** | **SD** | **Mean** | **SD** |
| Age |  |  | 51.4 | 13.6 | 53.1 | 18.1 |
| BMI |  |  | 30.2 | 8.4 | 28.0 | 7.4 |
| Comorbidities (of 15 presented) |  |  | 3.8 | 2.3 | 2.5 | 2.8 |
| *Some sets of percentages may not add up to 100% due to rounding. GED = General Educational Development (i.e., high-school equivalency test) SD = standard deviation* | | | | |  |  |
