# Supplementary material for: Expanding the purview of wellness indicators: validating a new measure that includes attitudes, behaviors, and perspectives
Source: Health Psychol Behav Med. 2021 Dec 1;9(1):1031–52. doi: 10.1080/21642850.2021.2008940 (PMC8648008; doi:10.1080/21642850.2021.2008940)
Supplement: Supplemental Material [file RHPB_A_2008940_SM8555.zip › DQ Wellness_Supp Table 2.docx]

| **Supplemental Table 2. Descriptive Statistics** | | | | | | |
| --- | --- | --- | --- | --- | --- | --- |
| Item | Minimum | Maximum | Mean | Std. Deviation | Skewness | Std. Error |
| Interested in activities | 0 | 4 | 2.48 | 1.10 | -0.30 | 0.04 |
| Feel like old self | 0 | 4 | 1.72 | 1.32 | 0.18 | 0.04 |
| Hard to find energy* | 0 | 4 | 1.95 | 1.30 | 0.10 | 0.04 |
| Zest for life | 0 | 4 | 2.08 | 1.25 | -0.05 | 0.04 |
| Able to feel joy | 0 | 4 | 2.53 | 1.15 | -0.32 | 0.04 |
| Think about the negatives* | 0 | 4 | 1.72 | 1.14 | 0.44 | 0.04 |
| Calm about the present | 0 | 4 | 2.03 | 1.19 | -0.03 | 0.04 |
| Sleep well | 0 | 4 | 1.85 | 1.30 | 0.02 | 0.04 |
| Chuckle at funny things in my day | 0 | 4 | 2.86 | 1.07 | -0.67 | 0.04 |
| Feel content | 0 | 4 | 2.47 | 1.13 | -0.33 | 0.04 |
| Take care of self | 0 | 4 | 2.32 | 1.19 | -0.20 | 0.04 |
| Don't let others affect mood | 0 | 4 | 2.03 | 1.12 | 0.04 | 0.04 |
| Notice beauty in world | 0 | 4 | 2.93 | 1.05 | -0.76 | 0.04 |
| Energy to be kind | 0 | 4 | 3.06 | 0.95 | -0.88 | 0.04 |
| So involved lose track of time | 0 | 4 | 1.98 | 1.20 | -0.02 | 0.04 |
