# Supplementary material for: Expanding the purview of wellness indicators: validating a new measure that includes attitudes, behaviors, and perspectives
Source: Health Psychol Behav Med. 2021 Dec 1;9(1):1031–52. doi: 10.1080/21642850.2021.2008940 (PMC8648008; doi:10.1080/21642850.2021.2008940)
Supplement: Supplemental Material [file RHPB_A_2008940_SM8555.zip › DQ Wellness_Supp Table 3.docx]

| **Supplemental Table 3: Exploratory Factor Analyses (EFA) using Geomin (Oblique) Rotating Loadings*** | | | | | | | | | | | | | | | | |
| --- | --- | --- | --- | --- | --- | --- | --- | --- | --- | --- | --- | --- | --- | --- | --- | --- |
| **One-Factor EFA** | |  | **Two-Factor EFA** | | |  | **Three-Factor EFA** | | | |  | **Four-Factor EFA** | | | | |
| **Item** | **Factor** |  | **Item** | **Factor** | |  | **Item** | **Factor** | | |  | **Item** | **Factor** | | | |
|  | **1** |  |  | **1** | **2** |  |  | **1** | **2** | **3** |  |  | **1** | **2** | **3** | **4** |
| Zest | **0.89** |  | Hard energy | **-0.77** | 0.23 |  | Zest | **0.86** | 0.08 | 0.00 |  | Zest | **0.79** | 0.23 | -0.07 | -0.01 |
| Content | **0.88** |  | Calm | **0.66** | 0.13 |  | Joy | **0.83** | 0.00 | 0.17 |  | Old Self | **0.69** | 0.03 | 0.18 | 0.00 |
| Joy | **0.88** |  | Old Self | **0.66** | 0.18 |  | Interested | **0.65** | 0.24 | -0.01 |  | Interested | **0.66** | 0.15 | 0.06 | 0.05 |
| Interested | **0.82** |  | Think negative | **-0.66** | 0.06 |  | Chuckle | **0.59** | 0.00 | 0.35 |  | Take care | **0.56** | -0.03 | 0.02 | 0.27 |
| Old Self | **0.78** |  | Sleep | **0.65** | 0.01 |  | Old Self | **0.54** | 0.37 | -0.11 |  | Chuckle | -0.02 | **0.80** | 0.03 | 0.06 |
| Chuckle | **0.75** |  | Interested | **0.51** | **0.37** |  | Content | **0.53** | 0.31 | 0.22 |  | Content | 0.13 | **0.67** | 0.28 | 0.01 |
| Beauty | **0.74** |  | Chuckle | 0.00 | **0.80** |  | Think negative | 0.04 | **-0.67** | 0.00 |  | Joy | 0.40 | **0.60** | 0.00 | -0.04 |
| Calm | **0.73** |  | Beauty | 0.00 | **0.79** |  | Hard energy | -0.02 | **-0.63** | 0.16 |  | Think negative | 0.03 | -0.27 | **-0.57** | 0.00 |
| Take care | **0.67** |  | Joy | 0.25 | **0.70** |  | Sleep | 0.09 | **0.60** | 0.00 |  | Kind | -0.03 | 0.38 | 0.03 | **0.55** |
| Kind | **0.67** |  | Kind | 0.02 | **0.68** |  | Others affect mood | -0.01 | **0.58** | 0.18 |  | Beauty | 0.04 | 0.47 | -0.03 | 0.48 |
| Sleep | **0.61** |  | Content | **0.38** | **0.57** |  | Calm | 0.25 | **0.55** | 0.01 |  | Others affect mood | 0.26 | 0.01 | 0.21 | 0.36 |
| Others affect mood | **0.60** |  | Zest | **0.42** | **0.54** |  | Kind | 0.00 | 0.43 | **0.54** |  | Sleep | 0.37 | -0.01 | 0.31 | 0.16 |
| Think negative | **-0.55** |  | Lose track of time | -0.15 | **0.49** |  | Beauty | 0.20 | 0.31 | **0.53** |  | Lose track of time | 0.14 | 0.22 | -0.19 | 0.15 |
| Hard energy | **-0.50** |  | Take care | **0.41** | **0.31** |  | Lose track of time | 0.31 | -0.11 | 0.23 |  | Calm | 0.27 | 0.27 | 0.40 | 0.02 |
| Lose track of time | 0.31 |  | Others affect mood | **0.44** | 0.21 |  | Take care | 0.32 | 0.36 | 0.10 |  | Hard energy | -0.29 | 0.03 | -0.44 | 0.02 |
| **Model Fit Statistics** |  |  |  |  |  |  |  |  |  |  |  |  |  |  |  |  |
| **RMSEA** | 0.106 |  |  | 0.092 |  |  |  | 0.078 |  |  |  |  | 0.068 |  |  |  |
| **90 Percent C.I.** | 0.10, 0.11 |  |  | 0.09, 0.10 |  |  |  | 0.07, 0.09 |  |  |  |  | 0.06, 0.08 |  |  |  |
| **Prob. RMSEA <= .05** | 0.000 |  |  | 0.000 |  |  |  | 0.000 |  |  |  |  | 0.000 |  |  |  |
| **CFI** | 0.959 |  |  | 0.974 |  |  |  | 0.984 |  |  |  |  | 0.991 |  |  |  |
| **TLI** | 0.952 |  |  | 0.964 |  |  |  | 0.974 |  |  |  |  | 0.981 |  |  |  |
| ** Items re-ordered by factor-loading magnitude to highlight factor structure* | | | | | | | |  |  |  |  |  |  |  |  |  |
