# Supplementary material for: Expanding the purview of wellness indicators: validating a new measure that includes attitudes, behaviors, and perspectives
Source: Health Psychol Behav Med. 2021 Dec 1;9(1):1031–52. doi: 10.1080/21642850.2021.2008940 (PMC8648008; doi:10.1080/21642850.2021.2008940)
Supplement: Supplemental Material [file RHPB_A_2008940_SM8555.zip › DQ Wellness_Supp Table 4.docx]

| **Supplemental Table 4. DQ Wellness Marginal Slopes, Intercepts, and Thresholds** | | | | | | | | | |
| --- | --- | --- | --- | --- | --- | --- | --- | --- | --- |
| **Item** | **Marginal Slope** | **Intercepts** | | | | **Thresholds** | | | |
|  |  | **c1** | **c2** | **c3** | **c4** | **b1** | **b2** | **b3** | **b4** |
| Interested in activities | 2.63 | 6.45 | 3.81 | 1.02 | -1.54 | -2.45 | -1.45 | -0.39 | 0.59 |
| Feel like old self | 2.27 | 3.41 | 1.84 | -0.12 | -2.09 | -1.51 | -0.81 | 0.05 | 0.92 |
| Zest for life | 3.15 | 5.05 | 2.52 | -0.11 | -2.69 | -1.60 | -0.80 | 0.04 | 0.85 |
| Able to feel joy | 3.51 | 7.50 | 4.22 | 1.25 | -1.69 | -2.14 | -1.20 | -0.36 | 0.48 |
| Calm about the present | 1.99 | 4.02 | 2.19 | 0.01 | -1.94 | -2.02 | -1.10 | 0.00 | 0.98 |
| Sleep well | 1.24 | 2.72 | 1.58 | 0.09 | -1.51 | -2.19 | -1.27 | -0.08 | 1.22 |
| Chuckle at funny things in my day | 2.05 | 5.38 | 3.02 | 1.18 | -1.01 | -2.62 | -1.47 | -0.58 | 0.49 |
| Feel content | 3.99 | 7.77 | 4.44 | 0.87 | -2.75 | -1.95 | -1.11 | -0.22 | 0.69 |
| Take care of self | 1.58 | 4.17 | 2.37 | 0.54 | -1.15 | -2.64 | -1.50 | -0.34 | 0.73 |
| Don't let others affect mood | 1.18 | 3.55 | 1.67 | -0.07 | -1.74 | -3.01 | -1.41 | 0.06 | 1.47 |
| Notice beauty in world | 1.93 | 5.01 | 2.87 | 0.99 | -1.14 | -2.60 | -1.49 | -0.51 | 0.59 |
| Energy to be kind | 1.45 | 4.87 | 2.95 | 1.18 | -0.93 | -3.35 | -2.03 | -0.81 | 0.64 |
| So involved lose track of time | 0.52 | 2.07 | 0.78 | -0.56 | -1.92 | -4.02 | -1.52 | 1.10 | 3.73 |
| Hard to find energy* | 0.86 | 2.15 | 1.06 | -0.17 | -1.55 | -2.50 | -1.23 | 0.20 | 1.81 |
| Think about the negatives* | 1.18 | 3.06 | 1.63 | 0.25 | -2.07 | -2.60 | -1.38 | -0.21 | 1.76 |
