# Supplementary figures and images for: Expanding the purview of wellness indicators: validating a new measure that includes attitudes, behaviors, and perspectives
Source: Health Psychol Behav Med. 2021 Dec 1;9(1):1031–52. doi: 10.1080/21642850.2021.2008940 (PMC8648008; doi:10.1080/21642850.2021.2008940)

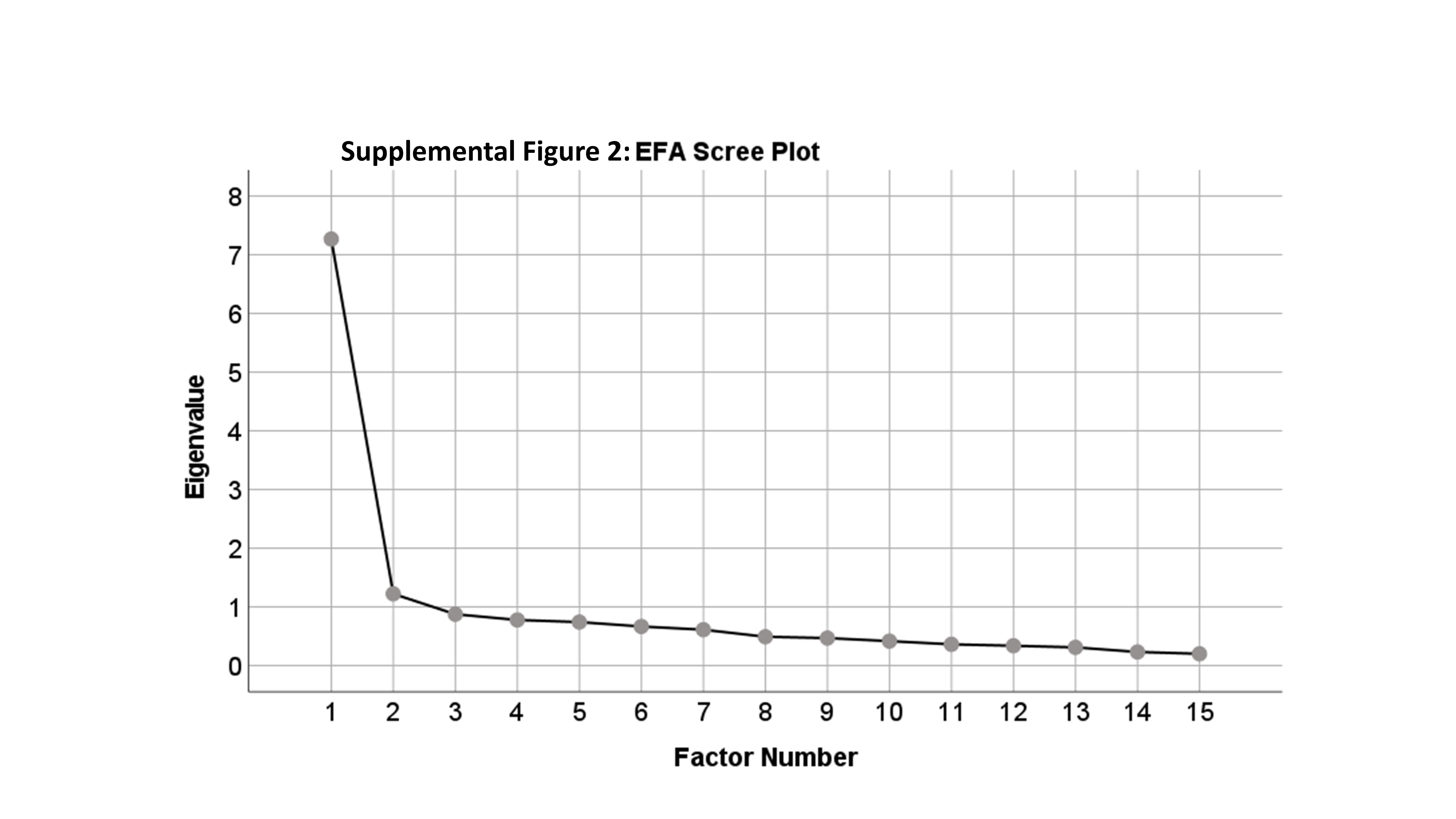

Supplement: Supplemental Material [file RHPB_A_2008940_SM8555.zip › DQ Wellness Supp. Figure 2.png]

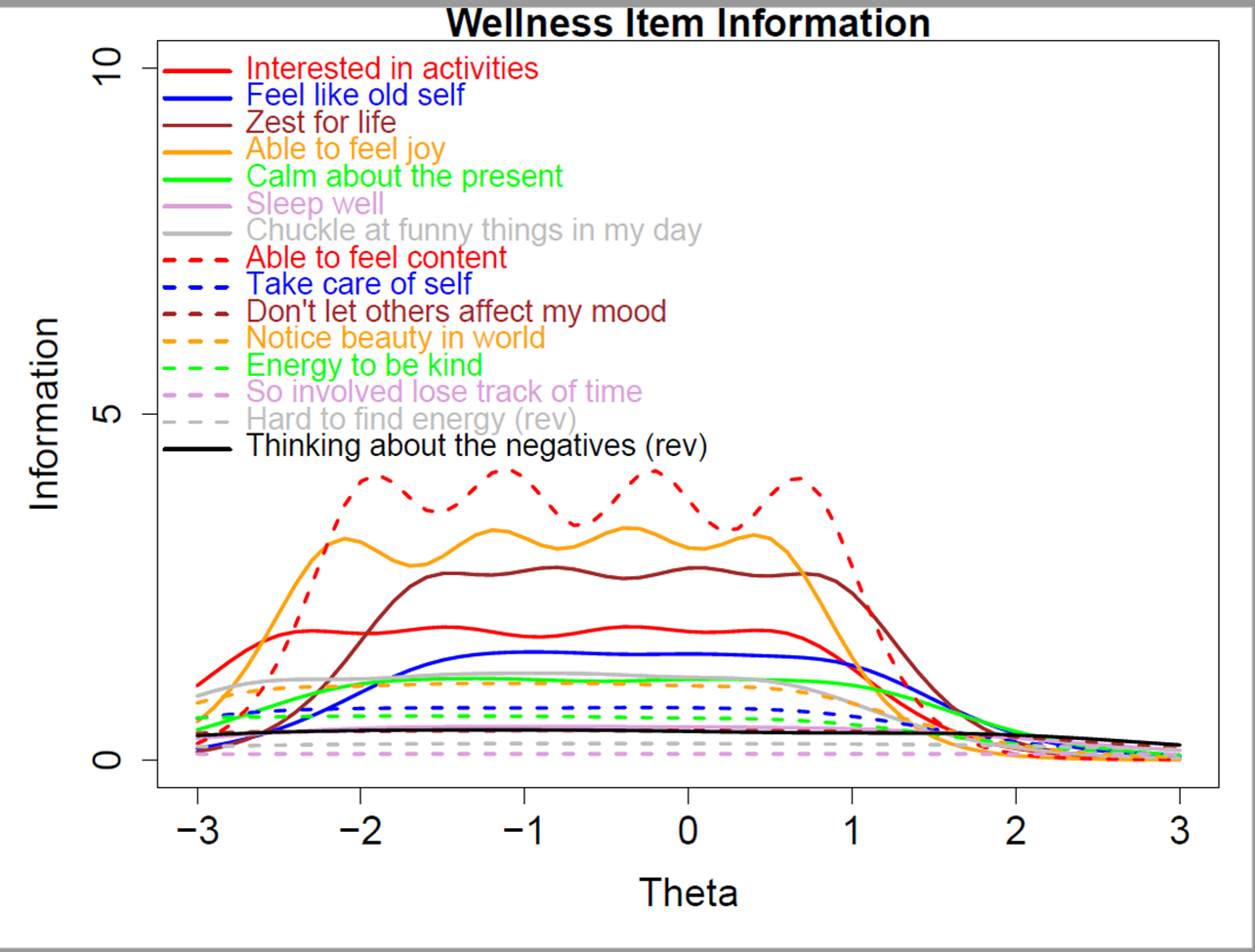


**Supplemental Figure 3_IRT Plots**


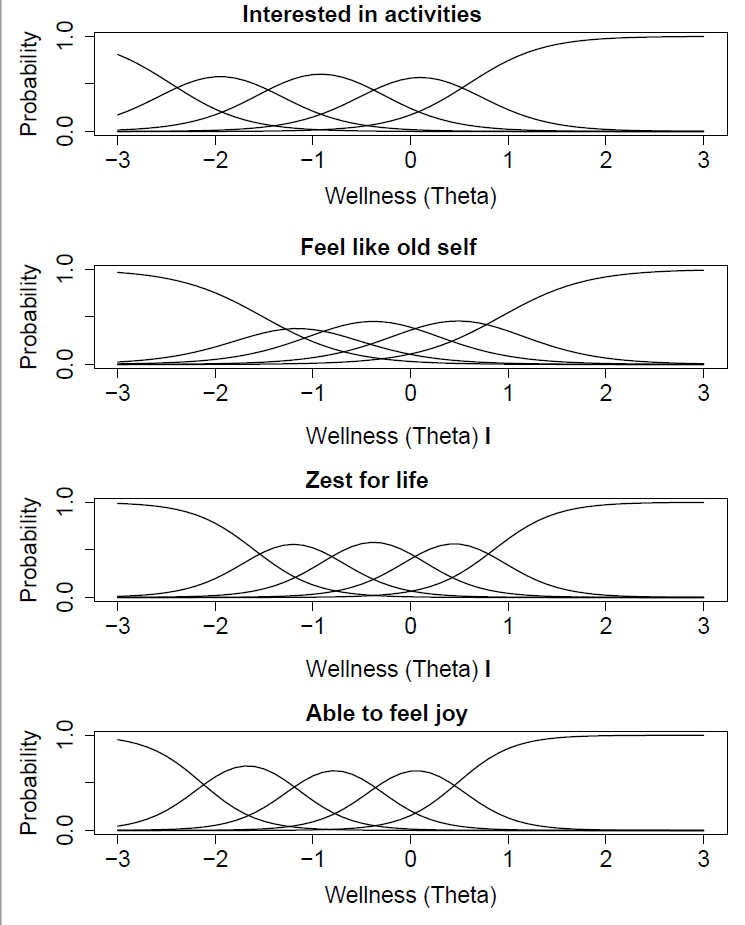


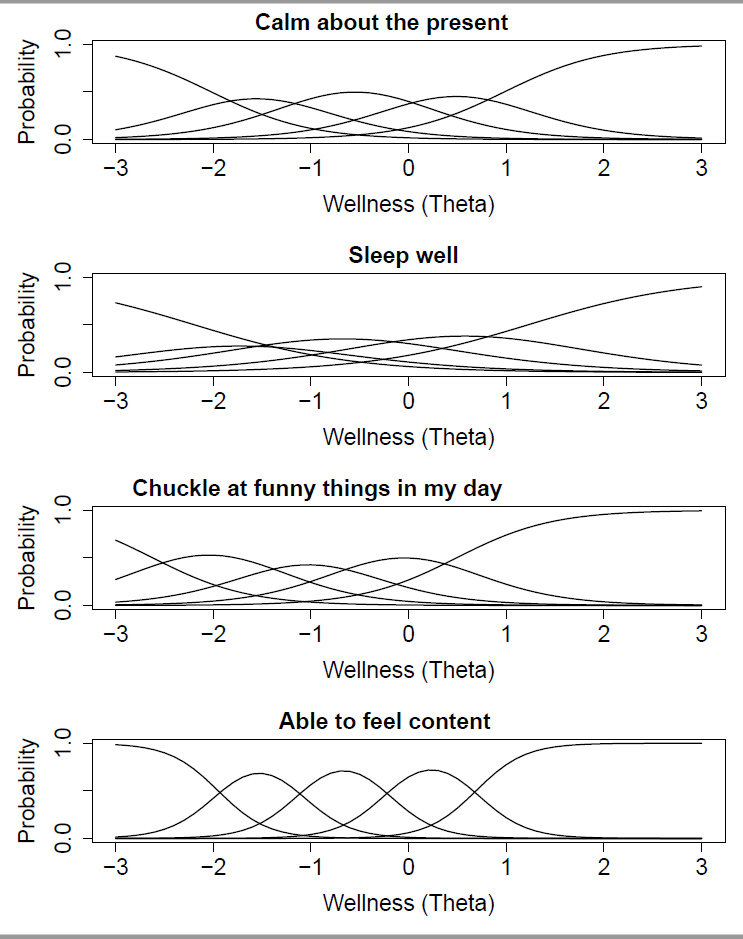


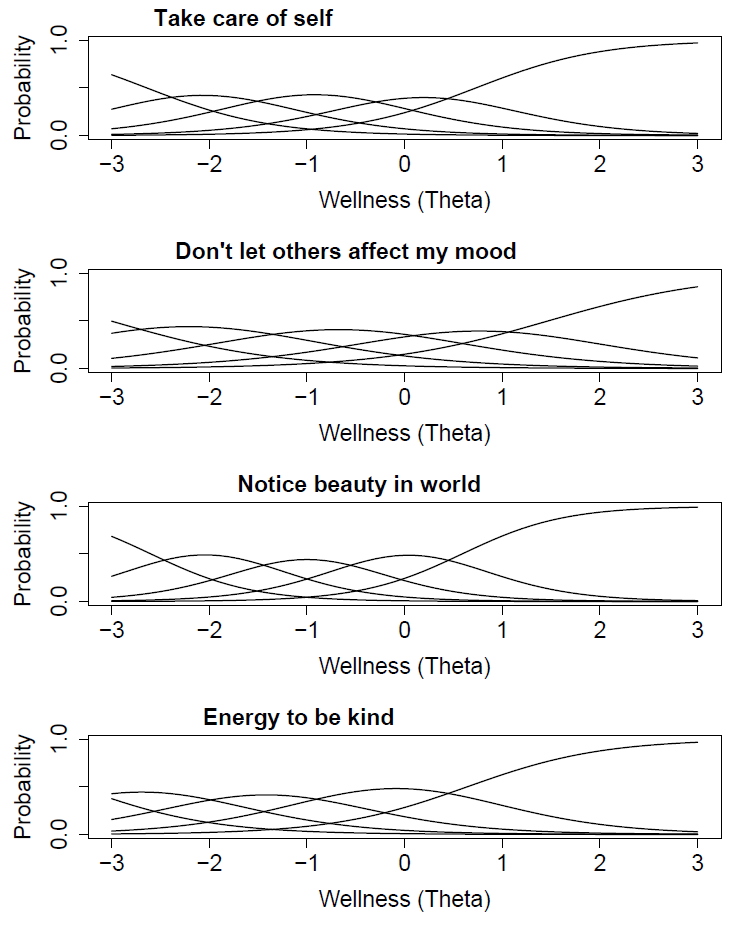


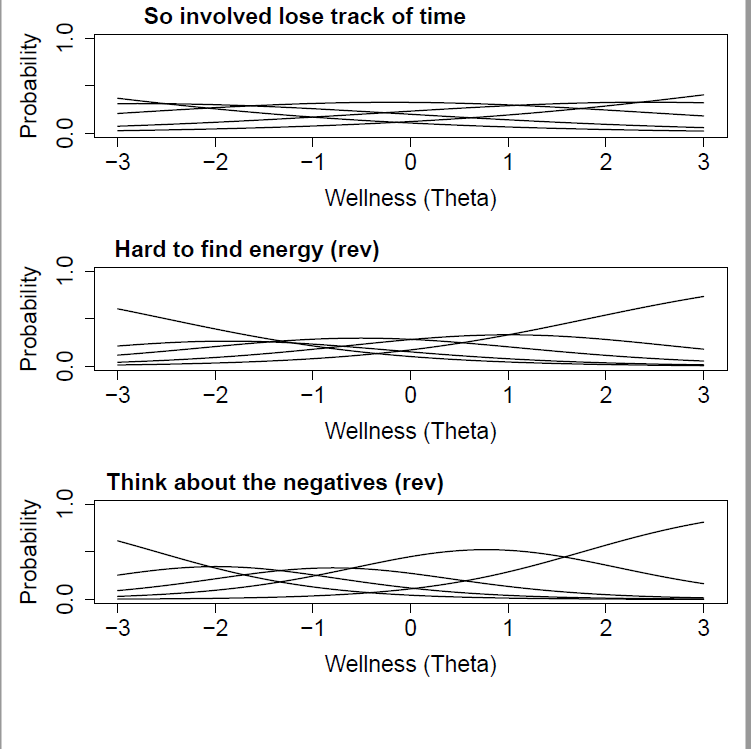

Supplement: Supplemental Material [file RHPB_A_2008940_SM8555.zip › DQ Wellness_SuppFigure3_IRT Plots.docx]

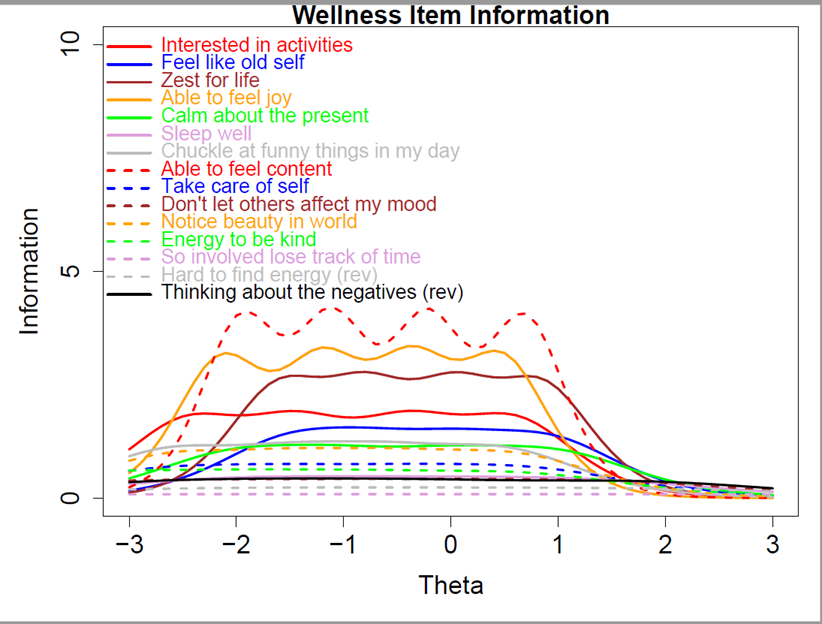

Supplement: Supplemental Material [file RHPB_A_2008940_SM8555.zip › SuggFigure3a_Item Information Function.png]
